# Artificial Intelligence in Coloproctology: A Review of Emerging Technologies and Clinical Applications

**DOI:** 10.3390/jcm13195842

**Published:** 2024-09-30

**Authors:** Joana Mota, Maria João Almeida, Miguel Martins, Francisco Mendes, Pedro Cardoso, João Afonso, Tiago Ribeiro, João Ferreira, Filipa Fonseca, Manuel Limbert, Susana Lopes, Guilherme Macedo, Fernando Castro Poças, Miguel Mascarenhas

**Affiliations:** 1 Precision Medicine Unit, Department of Gastroenterology, São João University Hospital, 4200-427 Porto, Portugal; joanamfmota8@gmail.com (J.M.); maj.almeida.14@gmail.com (M.J.A.); miguelpedro96@gmail.com (M.M.); francisco.cnm@gmail.com (F.M.); pedromarilio@hotmail.com (P.C.); joaoafonso28@gmail.com (J.A.); tiagofcribeiro@outlook.com (T.R.); su.isa.lopes@gmail.com (S.L.); guilhermemacedo59@gmail.com (G.M.); 2 WGO Gastroenterology and Hepatology Training Center, 4200-047 Porto, Portugal; 3 Department of Mechanical Engineering, Faculty of Engineering, University of Porto, 4200-065 Porto, Portugal; j.ferreira@fe.up.pt; 4 DigestAID—Digestive Artificial Intelligence Development, Rua Alfredo Allen n.° 455/461, 4200-135 Porto, Portugal; 5 Instituto Português de Oncologia de Lisboa Francisco Gentil (IPO Lisboa), 1099-023 Lisboa, Portugal; filipa.arfonseca@gmail.com (F.F.); manuellimbert@gmail.com (M.L.); 6 Artificial Intelligence Group of the Portuguese Society of Coloproctology, 1050-117 Lisboa, Portugal; castro.pocas@sapo.pt; 7 Faculty of Medicine, University of Porto, 4200-047 Porto, Portugal; 8 Department of Gastroenterology, Santo António University Hospital, 4099-001 Porto, Portugal; 9 Abel Salazar Biomedical Sciences Institute (ICBAS), 4050-313 Porto, Portugal

**Keywords:** artificial intelligence, coloproctology, colonoscopy, high-resolution anoscopy, anorectal manometry

## Abstract

Artificial intelligence (AI) has emerged as a transformative tool across several specialties, namely gastroenterology, where it has the potential to optimize both diagnosis and treatment as well as enhance patient care. Coloproctology, due to its highly prevalent pathologies and tremendous potential to cause significant mortality and morbidity, has drawn a lot of attention regarding AI applications. In fact, its application has yielded impressive outcomes in various domains, colonoscopy being one prominent example, where it aids in the detection of polyps and early signs of colorectal cancer with high accuracy and efficiency. With a less explored path but equivalent promise, AI-powered capsule endoscopy ensures accurate and time-efficient video readings, already detecting a wide spectrum of anomalies. High-resolution anoscopy is an area that has been growing in interest in recent years, with efforts being made to integrate AI. There are other areas, such as functional studies, that are currently in the early stages, but evidence is expected to emerge soon. According to the current state of research, AI is anticipated to empower gastroenterologists in the decision-making process, paving the way for a more precise approach to diagnosing and treating patients. This review aims to provide the state-of-the-art use of AI in coloproctology while also reflecting on future directions and perspectives.

## 1. Introduction

Coloproctology is a medical field that encompasses disorders of the rectum, colon, and anus. These diseases encompass significant morbidity and mortality; therefore, efforts have been made to leverage artificial intelligence (AI) to assist in the diagnosis, treatment, and follow-up of these disorders.

Coloproctology, as a field with strong imaging and diagnostic emphasis, greatly benefits from AI applications; therefore, its integration is growing exponentially, allowing for a more precise and personalized approach with higher availability and lower rates of medical errors [1]. AI is being utilized to develop more efficient technology for the diagnosis and management of colorectal carcinoma (CRC), inflammatory bowel disease (IBD), anorectal dysfunctions, and other anorectal pathologies.

AI systems can mitigate humans’ limitations due to visual perception, fatigue, distraction, and alertness, recently showing immense potential for assisting medical doctors and becoming a routine practice in many fields [1].

Therefore, the application of AI in healthcare, including gastroenterology, is anticipated to transform this field by enhancing diagnostic accuracy and reproducibility [2,3]. Indeed, standardizing lesion characterization and classification allows for a more objective and reproducible endoscopic assessment [4]. Therefore, a lot of research is being performed to enhance our understanding of AI in these different technologies. However, the rapid growth of these technologies brings ethical challenges that need to be addressed before widespread implementation in medical practice [5].

**Figure 1:**
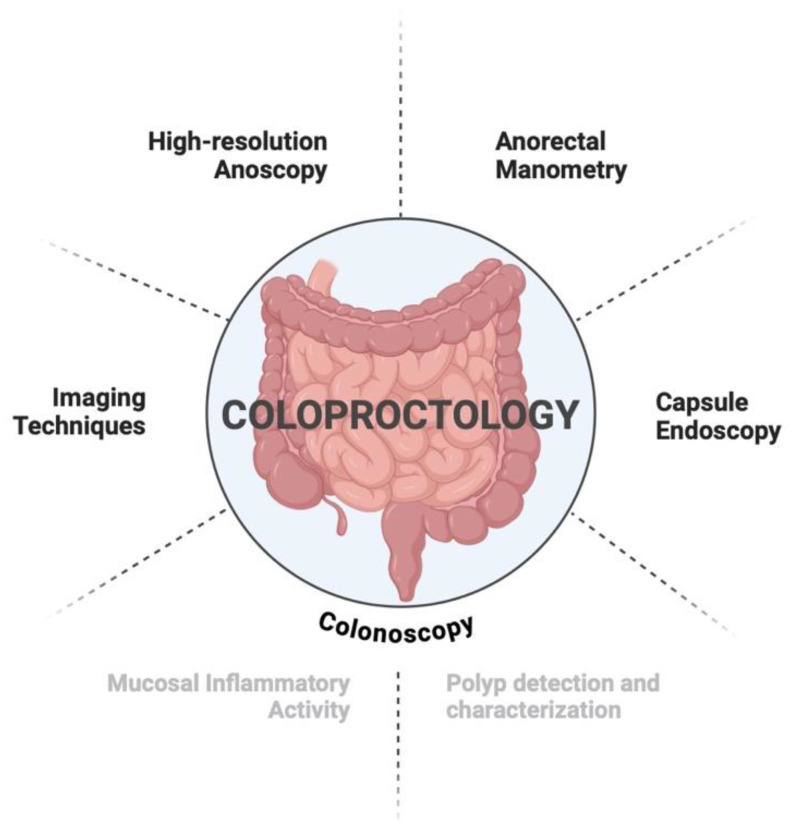
Study organization by diagnostic and therapeutic techniques used in coloproctology.

This review aims to provide a comprehensive overview of how AI is currently being implemented in coloproctology (Figure 1). It will reflect on future directions and perspectives, addressing both the limitations and potential uses of AI in this field.

## 2. State-of-the-Art

### 2.1. Colonoscopy

One of the most mature applications of AI in gastroenterology is in colonoscopy practice, where intense research has been going on over the last two decades, and AI-powered medical devices are already commercially available on the global market. Computer-aided detection (CADe) and computer-aided diagnosis (CADx) systems are probably the major uses of those AI technologies, aiming to improve the detection and characterization of polyps during colonoscopy. Initially, there was a focus on the detection of protuberant lesions such as polyps and tumors. Nowadays, the recognition that AI is a valuable tool in various areas has led to a significant rise in AI research focusing on its application in IBD. Most research studies concentrate on automating endoscopic scoring and predicting histological disease in real time.

#### 2.1.1. Polyp Detection and Characterization

##### CADe

Colorectal carcinoma (CRC) is one of the most preventable forms of cancer if colorectal polyps are detected and removed early in the mutational cascade. Therefore, the value of screening colonoscopy in reducing CRC is well established. Adenoma detection rates (ADRs) are highly operator-dependent, with studies reporting a wide range of 7% to 53% among endoscopists [6]. Missing neoplastic lesions might occur due to failure to recognize a lesion on the endoscopic image or incomplete exposure of the entire colorectal mucosa and is associated with the development of interval CRC, which represents 10% of all CRCs diagnosed [7]. There is a recognized polyp miss rate of 26% for adenomas, according to a recent systematic review and meta-analysis of colonoscopy studies [8]. Therefore, the ADR has become a critical quality indicator for an effective colonoscopy, and, alongside the endoscope’s technical role, the individual factors of endoscopists are certainly influential. Hence, AI integration into colonoscopy has been proposed as a tool to improve colonoscopic ADRs and possibly reduce the incidence of interval CRC.

CADe are deep learning (DL) algorithms tailored to support endoscopists by visually indicating potential polyps on the monitor in real-time settings. Several CADe systems have been developed, with some already clinically implemented, such as GI Genius (Medtronic, Ireland), which was introduced in 2019. Since then, there has been a growing interest in developing and demonstrating the efficiency of such devices. Several published articles, including meta-analyses and prospective RCTs, mainly retrieved from Asian populations, validated a sustained increase in ADRs using CADe systems compared to standard colonoscopy [9,10,11,12,13,14,15,16,17,18,19,20]. This benefit of CADe in improving the ADR seems to be replicated even with expert endoscopists with higher baseline ADRs [11]. Hence, CADe offers promise to minimize undesired operator-dependent variability in colonoscopy performance while also providing tools for further technical refinement for less experienced endoscopists. Additionally, considering the fact that these systems’ performance is also hindered by complete mucosal exposure, the combination of devices for mucosal exposure, such as endoCuff with CADe, reduces exposure errors and significantly improves colonoscopic quality outcomes and effectiveness [21].

A general criticism of CADe devices is related to false-positive activations that alert endoscopists to areas that would not normally warrant their attention, particularly to diminutive polyps (<5 mm) with minimal malignant potential. This could lead to unnecessary polypectomies and lengthy withdrawal times, jeopardizing the effectiveness of CADe systems. Nevertheless, recent studies suggested that the impact is negligible and that trained endoscopists are able to quickly dismiss most false-positive activations with no apparent negative effect on colonoscopy duration [22].

##### CADx

With the growing availability of different devices and technologies, the detection of diminutive polyps (<5 mm) has dramatically increased, representing 60% of polyps identified during colonoscopy. These polyps rarely progress to advanced adenomatous histology, and therefore, their malignant potential is minimal [23]. To date, the current practice is to remove all polyps seen for histological analysis, which places increased burden costs and workload on the entire clinical chain, from endoscopists to histopathological physicians. This has prompted cost-saving strategies aimed at improving the optical diagnosis of diminutive colorectal polyps, such as the PIVI document by the American Society for Gastrointestinal Endoscopy (ASGE), published in 2011. Strategies of “resect and discard” (resected without histological evaluation) and “diagnose and leave” (not resected) were to be applied pending specific thresholds for quality directives defined by ASGE [24]. Regrettably, many years have passed since the proposal of these strategies, and the implementation has been insufficient, mainly due to concerns about medico–legal implications, miscalculating endoscopic surveillance intervals, and a lack of proper training in optical diagnosis.

In light of the above-mentioned difficulties, the optical diagnosis of diminutive polyps remains a promising opportunity for the application of a reliable AI system for CADx.

CADx refers to the computer-assisted optical diagnosis of colorectal polyps using image analysis algorithms, classifying them as either neoplastic or non-neoplastic lesions [25,26,27]. The ability to accurately achieve that optical classification without histological analysis could allow institutions to adopt the aforementioned strategies of “resect and discharge” and “diagnose and leave” during colonoscopy [28,29,30,31,32]. In fact, based on recently published findings, CADx systems could reduce the proportion of polypectomies and pathology costs by more than 80% [33].

CADx has been validated not only in standard white-light imaging but also using different imaging enhancement modalities, such as narrow band ligation (NBI). In both cases, the optimal performance of CADx is sufficiently high and meets the PIVI criteria required to implement the cost-saving approach previously mentioned [22,27,34].

Despite the primary interest in the optical diagnosis of diminutive polyps, the appeal of CADx systems is manifold. Indeed, a more novel application of AI in colonoscopy is the prediction of the depth of invasion in colorectal lesions, which has a tremendous impact on prognosis and treatment decisions (endoscopic resection versus surgery). Current classifications using NBI, such as the Japan NBI Expert Team (JNET) and NBI International Colorectal Endoscopic (NICE) classifications, rely on the lesion’s color, vascular, and surface pattern to predict the depth of invasion, but their performance is highly operator dependent [35]. Applying AI to compute the prediction of submucosal depth invasion from an endoscopic image stands as a striking opportunity as it can be performed during the index endoscopy and is less reliant on the expertise of endoscopists. A recently published meta-analysis, including ten studies, compared the performance of CAD algorithms and endoscopists in predicting invasion depth in early CRC. Japan/Korea-based studies presented AUC, sensitivity, and specificity of 0.89, 62%, and 96%, respectively, while China-based studies had a pooled AUC, sensitivity, and specificity of 0.94, 88%, and 88%. According to data from Japanese, Korean, and Chinese studies, a positive result from a CAD system would increase the probability of the lesion being endoscopically unresectable to 83% and 68%, respectively [36].

Even though CADe and CADx are typically discussed separately, the ideal CAD system would integrate both concepts into a comprehensive workflow able to simultaneously detect and characterize polyps in real-time settings. Keeping that in mind, the initial AI systems enabling both functions are now under development, with some studies portraying promising results [37,38,39].

#### 2.1.2. Inflammatory Bowel Disease

Nowadays, the aim of the current strategy for IBD treatment is a treat-to-target approach that allows for the maximization of long-term results through tight disease control [40]. We know that a clinical physician’s experience influences the accuracy of differential diagnoses; AI could potentially reduce this inter-observer evaluation [41].

##### Mucosal Activity

In 2003, Sasaki et al. created the first-ever study using AI in IBD. The researchers aimed to develop a computer-aided grading system able to quantitatively evaluate mucosal activity using the Matts score, achieving high-performance results. This study was important because it showed the potential for creating an objective tool with low inter-observer variability for assessing the severity of ulcerative colitis (UC) with a high degree of sensitivity and specificity [42].

Later, Ozawa et al. developed a convolutional neural network (CNN) algorithm capable of assessing endoscopic disease severity in patients with UC using the Mayo endoscopic score. The CNN was trained using 26,304 colonoscopy images from 841 patients with ulcerative colitis (UC) and validated its performance of the CNN with 3981 images from 114 UC patients. The performance in identifying Mayo 0 vs. Mayo 0–1 was calculated. The CNN achieved an area under the curve (AUROC) of 0.86 and 0.98 to identify Mayo 0 and 0–1, respectively. The results were higher in the rectum [43]. Other studies in this field were made in 2021; Becker et al. also developed a DL able to predict a Mayo Clinic subscore from raw colonoscopy videos, achieving great performance with AUROCs of 0.84 for Mayo ≥ 1 and 0.85 for ≥2 and ≥3 [44].

Takenaka et al. developed a deep neural network (DNN) for assessing histologic and endoscopic remission using endoscopic images and biopsies from UC patients. The DNN was constructed using 40,758 images from colonoscopies and 6885 biopsy results and the model was validated using 4187 endoscopic images and 4104 biopsies. Achieving excellent results, the model detected endoscopic remission with an accuracy of 90.1% and histologic remission with an accuracy of 92.9%. This study proved the potential of AI in helping identify patients in remission without biopsies [45]. Later, the same group adapted their previous DNN to full video colonoscopy and evaluated its validity in real-time detection of histological mucosal inflammation. They performed a multicenter prospective study whose aim was to compare DNN histologic assessments (healing or active) with pathologists’ evaluations. The second endpoint was to evaluate the ability of the DNN to determine UC severity compared to IBD experts using endoscopic images. Achieving excellent results, the DNN identified histological inflammation in 729 (81%) of the biopsies specimens and predicted histological remission with a sensitivity of 97.9%, a specificity of 94.6%, a positive predictive value of 98.6%, and a negative predictive value of 92.1% [46], reinforcing the potential of reducing the number of biopsies needed. Real-time histological assessment is made possible by ultra-high-definition imaging modalities such as confocal laser endomicroscopy and endocytoscopy [47]. Maeda et al. developed a computer-aided diagnosis system able to predict persistent histologic inflammation using endocytoscopy, achieving excellent results with diagnostic sensitivity, specificity, and accuracy of 74%, 97%, and 91%, respectively [48]. Other authors also developed AI models with the goal of predicting histology and the risk of flare. More recently, in 2023, Lacucci et al. aimed to distinguish histological remission/activity in biopsies of UC and predict flare-ups by developing a CNN [49]. The same group also developed a computerized image analysis system able to predict response to biologics in IBD by analyzing probe confocal laser endomicroscopy in vivo [50]. Many other studies aimed at automatic estimation of UC severity were made using AI models [51,52,53].

These studies provided, most likely, a tool for reducing intra- and inter-observer evaluation and potentially sparing economic and ecological resources.

Stidham et al. were the first to compare the performance of a DL model to that of human reviewers when evaluating the severity of endoscopic UC. The model achieved excellent performance for distinguishing remission from moderate-to-severe disease with an AUROC of 0.966, a PPV of 0.87, a sensitivity of 83.0%, a specificity of 96.0%, and an NPV of 0.94. The results were similar to the agreement between the reviewers [54].

In 2021, Gottlieb proved that a DL model can also predict the level of severity from full-length endoscopic videos [55]. A DL algorithm was also developed to classify the Mayo endoscopic subscore in patients with UC using still images from colonoscopy [56]. In 2023, Kim et al. also developed a DL model for distinguishing Mayo subscores 0 and 1, achieving an AUROC above 0.90 [57]. Sutton et al. tested several DL CNNs to distinguish UC from non-UC pathologies in the largest multi-class image and video dataset, achieving excellent results with the best accuracy of 87.5% and an AUC of 0.90 [4].

##### Differential Diagnosis of Colitis and Others

Despite the fact that there is already extensive research in the field of IBD, that evidence seems to be expanding in other aspects beyond the evaluation of mucosal inflammatory activity. Promising studies using AI to help correlate clinical data and colonoscopy findings, for example, to differentiate between surveillance and non-surveillance colonoscopies or to support decision systems [58,59,60]. Some studies use AI to assist endoscopists in the detection of neoplasias in IBD patients, achieving better results when compared to the experts [61]. Recently, new research aimed to develop an AI algorithm to distinguish IBD from other causes of colitis, such as infectious and ischemic colitis [59]. Ruan et al. developed a CNN to assist in the endoscopic diagnosis of IBD between Crohn’s Disease (CD) and UC. This study was multicenter with a large dataset (1772 patients with 49,154 colonoscopy images), achieving excellent results with less time consumed [62]. Wang et al. also developed a CNN to distinguish UC from CD. They used a large dataset (15,330 colonoscopy images from 217 CD patients, 279 UC patients, and 100 healthy subjects) and achieved a high overall accuracy of 92.04% in classifying CD, UC, or normal [63].

There have been several studies focusing on the use of AI to discriminate GI tuberculosis from CD. The latest systematic review on this subject concluded that AI is of added value in these cases, especially when combining various data (endoscopic, radiological, and laboratory findings) [64,65,66].

Other research studies were made to create CNNs able to diagnose various GI diseases (UC, esophagitis, and a healthy colon) using endoscopy images, achieving high performance with accuracies of 99.50% and 99.16% on the validation and test sets. The results of this study reinforce the application of AI to improving diagnostic accuracy in GI diseases [67]. Other studies were created with the goal of using AI for real-time differentiation between IBD and other intestinal diseases [68]. Also, a CNN was developed to automatically exclude non-informative video frames like frames with motion blur, frames obscured by liquid or solid debris, frames when the camera is too close to the colon wall, and frames overexposed due to the failure of automatic lighting controls. These results were important for evaluating the ability of further colonoscopy video analysis [69].

### 2.2. High-Resolution Anoscopy

**Figure 2:**
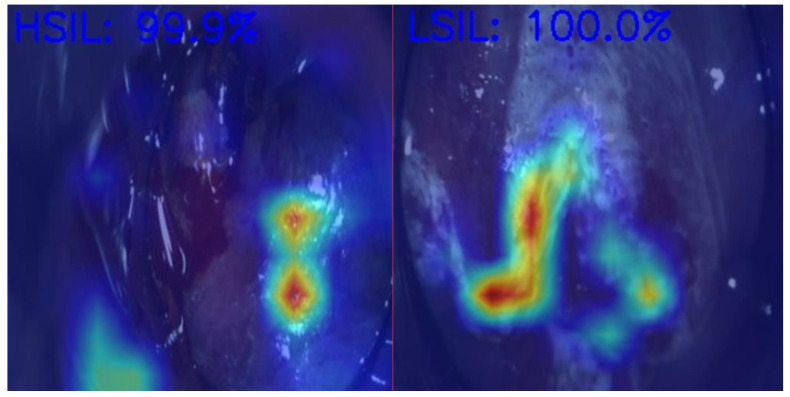
Artificial intelligence model predictions in differentiating an HSIL from an LSIL. HSIL—High-grade squamous intraepithelial lesion; LSIL—Low-grade squamous intraepithelial lesion. Image kindly provided by the working group of Precision Medicine Unir, CHUSJ/FMUP/FEUP.

Anal Squamous Cell Carcinoma (ASCC) is a growing concern among gastrointestinal malignancies, especially in HIV patients, the male homosexual population, and immunocompromised individuals, highlighting the importance of screening in those populations [70]. ASCC is preceded by dysplastic lesions with different malignant potentials, such as low-grade squamous intraepithelial lesions (LSILs) and high-grade squamous intraepithelial lesions (HSILs). Differentiating between these two entities is critical for treatment decisions, as LSILs can be managed expectantly, while HSILs require active treatment (ablative techniques or excisional procedures) to prevent progression to ASCC [71]. In Figure 2, we demonstrate an example of the use of AI in helping medical doctors differentiate HSILs from LSILs.

Screening high-risk populations for ASCC is broadly accepted despite inconsistent guidelines [72,73]. The available screening strategies include digital rectal examination, cytology, and high-resolution anoscopy (HRA), with the most robust evidence being HRA coupled with tissue sampling in the presence of cytological abnormalities [74,75].

HRA allows for a visual inspection of the transition zone, anal canal, and perianal skin under magnification. Together with the application of acetic acid and Lugol’s iodine, the goal is to render visible and prominent certain characteristic changes possibly representative of underlying dysplasia, therefore guiding subsequent biopsies or treatment decisions [76]. When accurately executed, HRA has the potential to diminish the risk of ASCC by nearly 60% [70]. Nevertheless, concerns remain regarding its cost-effectiveness as a screening method. HRA is associated with suboptimal specificity, a poor inter-observer agreement on visual impression, and a steeper learning curve, which in large part contributes to a shortage of skilled providers who perform HRA. As a result, in 2016, the International Anal Neoplasia Society issued guidelines for standardizing the practice of HRA, including training, lesion description, and quality criteria [77].

Through the automated analysis of patterns, AI algorithms have the potential to accelerate and standardize complex tasks, particularly beneficial to HRA. Indeed, HRA is one of the latest diagnostic methods in gastroenterology that is under study for the integration of AI technology. In 2022, the first deep learning method to detect and differentiate HSILs from LSILs in HRA frames was developed, and very promising results have already been achieved [78]. However, at that time, the influence of staining methods on the diagnostic performance of this type of AI was not taken into consideration. To finally address this gap, a subsequent deep learning system was developed for the automatic differentiation of HSILs from LSILs in HRA frames across various subsets. Those subsets encompassed non-stained, acetic acid, lugol iodine, and after manipulation (e.g., radiofrequency ablation, laser ablation, infrared coagulation, plasma coagulation, or surgical ablation). The global accuracy of the model was 98,8%, with 97% sensitivity and 99% specificity. In group subanalysis, the model consistently demonstrated high diagnostic performance metrics with overall accuracy varying between 92.7% in acetic acid and 98.1% in post-manipulation [79]. These findings not only revealed the robust performance in detecting ASCC precursor detection but also unveiled the potential to predict relapse following therapeutic interventions in the anal canal.

Nevertheless, despite the significant promise already achieved, these studies are preliminary, and future multi-centric and prospective research is needed to draw conclusions about broader populations.

### 2.3. Functional Studies—Anorectal Manometry

**Figure 3:**
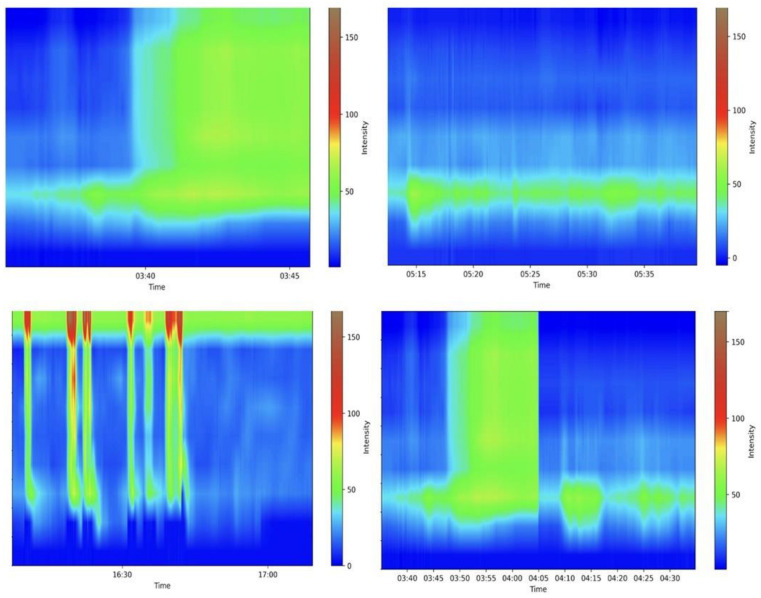
Examples of various anorectal maneuvers. Image kindly provided by the working group of Precision Medicine Unir, CHUSJ/FMUP/FEUP.

Anorectal manometry is the gold standard in diagnosing many anorectal disorders [80]. Indeed, this method evaluates several parameters involved in the processes of defecation and continence; a precise and specialized evaluation is needed, requiring experience from the medical doctor. Figure 3 shows some examples of the maneuvers evaluated. A Portuguese group pioneered the development of a machine learning (ML) model that accurately differentiated between fecal incontinence and obstructed defecation manometric patterns. They achieved remarkable results in all the evaluated outcomes, particularly in the gradient boost model, which achieved an area under the curve of 0.939, further highlighting the potential benefits of employing these algorithms in anorectal manometry [81]. Levy et al. generated three AI models (traditional ML, DL, and a hybrid approach) able to identify dyssynergic patterns using three-dimensional high-definition anal manometry. All models achieved high diagnostic performance metrics, but the DL algorithm approached ambiguous tests more cautiously than other models [82]. More studies are needed before these models can be applied in medical care.

### 2.4. Radiological Imaging Techniques

The integration of AI in medical imaging was one of the first applications of AI in medical fields. Its use in coloproctology is no exception, especially in the staging and further management of CRC. Indeed, the integration of AI in CRC imaging shows the potential to enhance diagnostic accuracy, tumor delineation, and lymph node assessment, among others [83,84].

In the management of Crohn’s disease, imaging techniques are essential, particularly for disease classification and activity assessment [85]. AI in this field is increasing; however, it is still diminished when compared to CRC screening. To assess disease activity, semi-automated models to identify and quantify disease activity in a reproducible manner were created [85,86,87].

## 3. Discussion

Coloproctology is a major domain within gastroenterology, where the rapid expansion and evolution of AI are revolutionizing healthcare through cutting-edge advancements in imaging analysis and pattern recognition.

Many advantages are pointed out by AI. First, it offers an unparalleled standardization of optical diagnosis performance in an operator-independent way, unaffected by the endoscopist’s training, fatigue, or distraction. It has a tremendous impact on training and competence assessments, providing valuable feedback to non-expert endoscopists and gastroenterologists. It may also automatically generate endoscopic reports, increasing not only the accuracy of the report itself but also standardizing lesion characterization while also saving time that would otherwise be spent writing the reports.

This review gives an overview of the landscape of AI application in coloproctology, ranging from its impact on CRC screening to its valuable role in the management of IBD, but also to its growing interest in less explored techniques such as anoscopy and anorectal manometry.

Regarding the application of AI in colonoscopy, the future goes beyond the detection and characterization of polyps, and new development opportunities are now turned to the standardization of colonoscopic quality parameters, such as bowel preparation [88,89,90], cecal intubation rates, withdrawal speed [17,18,91], scope-slipping alerts, and probably the most attractive quality parameter, the estimation of mucosal exposure [92]. It is desirable that the combination of CADe, CADx, and quality assurance tools result in an increase in the overall effectiveness and performance of colonoscopy, although much work needs to be performed in this area.

**Figure 4:**
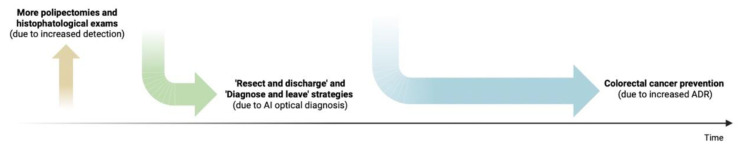
Healthcare-related costs over time with the application of AI in colonoscopy. Initially, these costs are expected to rise due to the upfront investment in equipment and increased detection rates, potentially leading to higher costs for conducting pathological studies. However, in the long run, these costs are expected to decrease, as greater diagnostic confidence may reduce the need for pretreatment biopsies.

The use of AI in healthcare generates some concerns that need to be addressed for its smoother application. Besides clinical effectiveness, health economic evaluation is another critical area to consider for AI implementation in clinical practice, particularly in colonoscopy, a well-established method for CRC screening. Recent studies suggest that the use of aided-colonoscopy systems might increase short-term healthcare costs by increasing the number of detected polyps, polypectomies, and histopathological exams, in addition to the cost of the AI system itself [93,94]. However, AI-optimized optical diagnosis, when used as an alternative to actual histopathological assessment with high confidence, may significantly reduce the need for pretreatment biopsies and lower colorectal cancer rates in the long term, ultimately resulting in a reduced carbon footprint and considerable healthcare cost savings (Figure 4).

Moreover, the more precise evaluation of inflammatory mucosal activity provides physicians with a valuable tool to better tailor treatments and reduces the need for frequent serial endoscopic evaluations. This approach not only portrays a more favorable economic paradigm but also improves patient outcomes by enabling more individualized treatment. However, large-scale studies with long-term follow-up are necessary to determine the economic and clinical benefits of these systems [5].

## 4. Conclusions

The future of AI in healthcare, particularly in coloproctology, looks remarkably promising as AI and coloproctology computer-aided devices continue to evolve and as further research validates their impact on endoscopic and healthcare outcomes. Ultimately, it will be the collaboration between AI technologies and medical expertise accumulated over the years that will guide us into a new era of enhanced diagnosis and improved patient care [95].
